# Is Premenstrual Syndrome Associated with Inflammation, Oxidative Stress and Antioxidant Status? A Systematic Review of Case–Control and Cross-Sectional Studies

**DOI:** 10.3390/antiox10040604

**Published:** 2021-04-14

**Authors:** Dominika Granda, Maria Karolina Szmidt, Joanna Kaluza

**Affiliations:** Institute of Human Nutrition Sciences, Warsaw University of Life Sciences—SGGW, 159C Nowoursynowska Str., 02-776 Warsaw, Poland; maria_szmidt@sggw.edu.pl (M.K.S.); joanna_kaluza@sggw.edu.pl (J.K.)

**Keywords:** premenstrual syndrome, menstruation, oxidative stress, inflammation, antioxidants, women, systematic review

## Abstract

Premenstrual syndrome (PMS) is a cyclically occurring combination of various symptoms, leading to decreased life quality among approximately 30% of women of childbearing age. PMS etiology remains unknown; however, there are some suggestions that inappropriate inflammatory response and oxidative stress are involved. This study aimed to systematically review case–control and cross-sectional studies investigating inflammation markers, oxidative stress, and antioxidant status among women with PMS and controls. The study protocol was registered with PROSPERO (no. CRD42020178545), and the authors followed the guidelines for performing a systemic review recommended by Preferred Reporting Items for Systematic Reviews and Meta-Analyses (PRISMA). By searching PubMed and Scopus databases (up to 8 January 2021), six case–control studies and five cross-sectional studies of medium or high quality were classified to the review. The systematic review included 652 women with PMS and 678 controls, for whom 36 eligible markers were determined. Limited evidence indicates increased levels of inflammatory parameters and suggests decreased antioxidant status in PMS women. Insufficient data with inconsistent results made it impossible to formulate a firm conclusion on the contribution of oxidative stress in PMS occurrence. To acknowledge the role of inflammation, oxidative stress, and antioxidant status in the pathophysiology of PMS, further research with case–control design and large study groups is needed.

## 1. Introduction

Premenstrual syndrome (PMS) is a combination of emotional and physical symptoms that occur cyclically in the luteal phase of the menstrual cycle and significantly influence the quality of life [1]. It is a global disorder, diagnosed in 12–48% of reproductive-age women with a great variety between countries [2,3]. Premenstrual dysphoric disorder (PMDD) is characterized by more severe symptoms than PMS; it is less common and occurs in 3–8% of women. Despite extensive research, the etiology of PMS and PMDD remains unknown, although some potential mechanisms to explain this issue have been proposed.

There are speculations that women with PMS are more sensitive to fluctuations in sex hormone levels during the menstrual cycle [4]; however, some studies have not shown differences in hormone levels between healthy and PMS women [5,6]. Another leading hypothesis is alternation in response to either withdrawal or exposure to allopregnanolone (a gamma-aminobutyric acid agonist and progesterone metabolite). It is proved that allopregnanolone production blockage reduces premenstrual symptoms, and one of the PMS pharmacotherapies is a serotonin reuptake inhibitor, which affects allopregnanolone levels [7,8].

Another potential mechanism currently under investigation is an inappropriate inflammatory response to various stimulants (for example, biological or physical) and the occurrence of oxidative stress defined as an imbalance between the production of reactive oxygen species (ROS) and their inactivation by antioxidant protection mechanisms [9]. Both inflammation and oxidative stress appear in the healthy human organism as an effect of metabolic activity; in some cases, inflammation helps cells to adapt and survive stress conditions [10]. However, chronic inflammation and oxidative stress can lead to many diseases [11], and they are also considered possible factors of PMS development [12,13]. It is known that women of reproductive age experience alternation of inflammation status during the menstrual cycle [14]. Until now, relatively many studies have been conducted in which individual biomarkers of inflammation and oxidative stress were assessed in the PMS context; however, although their results are inconsistent, no literature review has been performed to order and summarize them. Therefore, we conducted a systematic review to clarify potential associations of inflammation, oxidative stress, and antioxidant status with PMS.

## 2. Materials and Methods

The protocol for this review has been registered in the International Prospective Register of Systematic Reviews (CRD42020178545).

### 2.1. Literature Search

An initial systematic review of the literature was conducted in May 2020 with PubMed and Scopus, with an update in January 2021. Databases were searched separately by two authors (D.G., M.K.S.) to identify studies on inflammation, oxidative stress markers, and antioxidants levels in women with PMS and control groups. The systematic review had included studies published up to 8 January 2021. For searching the following key terms and the conjunctive normal form were used: ((premenstrual syndrome) OR (PMS)) AND ((inflammation) OR (DNA damage) OR (oxidative stress) OR (antioxidant capacity) OR (antioxidant status) OR (lipid peroxidation) OR (catalase) OR (glutathione peroxidase) OR (glutathione reductase) OR (8-isoprostane) OR (nitric oxide) OR (8-hydroxyguanosine) OR (superoxide dismutase) OR (malondialdehyde) OR (hs-CRP) OR (nitrotyrosine) OR (protein carbonyls) OR (deoxyguanosine) OR (interleukin-6) OR (4-hydroxynonenal) OR (protein carbonyl content) OR (advanced glycation end products) OR (advanced oxidation protein products)). We also performed a manual search of the retrieved articles’ references to identify other potentially eligible studies.

### 2.2. Inclusion and Exclusion Criteria

Inclusion criteria were as follows: (1) studies where women with PMS and healthy controls were compared for markers of inflammation or/and oxidative stress or/and antioxidants; (2) studies with the use of a well-described scale or specific criteria to diagnose PMS; (3) studies involving nonpregnant, currently menstruating women in a general good health condition; (4) studies of case–control or cross-sectional design; (5) studies in English. Studies were excluded if: (1) there was no control group; (2) there was no description of the scale or criteria used to diagnose PMS; (3) no inflammation or oxidative stress or antioxidant markers were examined; (4) the language of the study was other than English. Two authors (D.G., M.K.S.) applied these criteria, and any disagreements were discussed and resolved by a consensus or consultation with the senior author (J.K.). We decided not to include as one of the inclusion criteria a strict PMS diagnosis established by the Daily Symptom Rating due to the International Society for Premenstrual Disorders position, according to which it is necessary only for clinical trials and intervention studies [15]. Instead, we defined that the tool used to diagnose PMS should be validated or adequately described by the authors.

### 2.3. Data Extraction and Analysis

All identified studies were reviewed independently by two authors (D.G., M.K.S.), and results were entered into data tables designed before data extraction. The following data were extracted from the studies: authors’ names, study design, year of the publication, study location, number of cases and controls, diagnostic criteria for PMS, mean ± standard deviation (SD) or range of age for cases and controls, participants characteristics (i.e., health and physiological condition, medication), biomarkers of interests, and information whether the results were adjusted for potential confounders. Moreover, we extracted data concerning the main results of the studies included in the review: mean ± SD or median and range of biological parameters for cases and controls, units, type of biological sample, *p*-value between cases and controls.

### 2.4. Quality Assessment

The quality of the studies was assessed independently by two authors (D.G., M.K.S.) according to a modification of the Newcastle–Ottawa Quality Assessment Scale (NOS) separately for case–control [16] and for cross-sectional studies (each study type had its modification of NOS) [17] as presented in Table 1.

We used a modified version of NOS in order to include the specificity of the subject of the review; for example, for case–control studies we decided to award points not only for the validated tool (as in the original version) to assess PMS, but also for a well-described one, due to the reasons mentioned previously. Due to the fact that the potentially important factors contributing to PMS are not definitely defined, we assigned a point for including any of them (such as body mass index or age of menarche) in the adjustment of the study results. The NOS is recommended by the Cochrane Collaboration for use in nonrandomized studies, as a validated, quick and adaptable tool for quality assessment. However, it should be underlined that it is not validated for cross-sectional studies and some researchers indicate problems with its poor agreement in point awarding. The NOS evaluates three parameters (selection, comparability, and exposure or outcome depending on the type of the study) divided across eight (for case–control studies) or seven (for cross-sectional studies) specific items [16,17]. Detailed rules, according to which points were awarded in individual categories, are shown in Supplementary Materials.

## 3. Results

**Figure 1:**
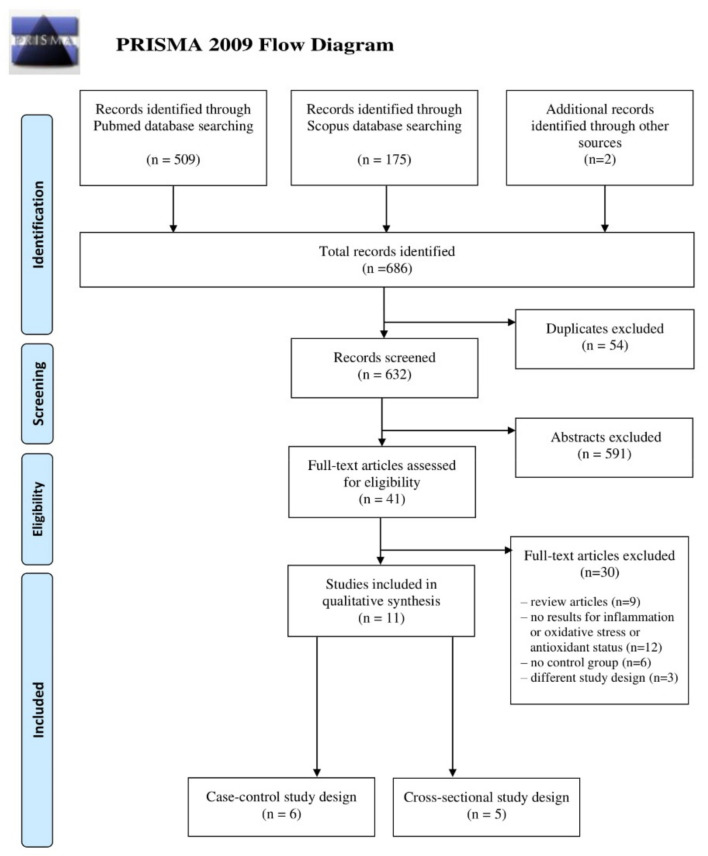
Literature review flow diagram of the selection process according to the Preferred Reporting Items for Systematic Reviews and Meta-Analyses (PRISMA) Statement.

Six hundred and eighty-four papers were identified in the initial databases search (509 for PubMed and 175 for Scopus) and two additional records were identified through the manual search of the reference lists of the eligible articles. The selection of papers for this review was performed according to the Preferred Reporting Items for Systematic Reviews and Meta-Analyses (PRISMA) flow chart—Figure 1.

Based on inclusion and exclusion criteria, 11 studies were selected and included in the systematic review: six with case–control and five with cross-sectional design. The characteristics of the studies included in this review are summarized in Table 2. Most identified papers were published after 2010 (with two exceptions from 2007 [18] and 2010 [23]). Six studies were conducted in Asia (four in Iran [20,25,26,28], one in Kuwait [24], and one in India [23]), three in Eastern Europe (Turkey [18,19,22]), and two in northern and South America (USA [27] and Brazil [21]). The sizes of studies were relatively small, with the number of PMS cases ranging from 11 [18] to 134 [25] and the number of controls from 10 [18] to 163 [28]. In the included studies, a wide variety of biomarkers were assessed; thus, we summarized and divided them into three main categories (inflammatory, oxidative stress, and antioxidant status parameters) with subgroups, as presented in Table 3.

The methodological quality assessment of the studies included in the review indicated that nearly 64% of the articles were of high quality [18,19,20,21,25,26,27] (67% of the case–control studies [18,19,20,21] and 60% of the cross-sectional [25,26,27]), while the remaining studies were of medium quality [22,23,24,28] (Table 1). Only in one study results were adjusted for potential confounders such as age, body mass index, or smoking status [27].

### 3.1. Inflammatory Biomarkers

Among 11 studies included in the systematic review, four (three of high [21,25,27] and one of medium [24] quality) examined inflammatory biomarkers, and most of them assessed interleukins concentration (Table 4). In three independent studies, PMS cases had higher levels of interleukin IL-1β [21,24,27] and IL-8 [21,24,27] compared to controls; the IL-1β level was statistically significantly higher in urine in one high-quality study [21], and in two other studies, non-significantly higher serum concentrations were found [24,27]. The IL-8 was statistically significantly higher in cases than controls in two studies (one in serum [24] and one in urine [21]). Each of IL-4 [24,27], IL-7 [21,27], and IL-10 [21,27] were analyzed in two studies. Bertone-Johnson et al. [27] found statistically significantly higher IL-4 and IL-10 concentrations in PMS cases’ serum than in controls. Moreover, for IL-4, a similar tendency in serum was found in a study conducted by Azizieh et al. [24], while Foster et al. [21] did not find a difference in urine IL-10 concentration between PMS cases and controls. A statistically significantly higher concentration of IL-7 in PMS women’s urine compared to the controls was found by Foster et al. [21], and a similar tendency in serum was observed by Bertone-Johnson et al. [27]. Other interleukins, such as IL-2, IL-5, IL-6, IL-12, IL-13, and IL-17, were determined in serum only in single studies. A significantly higher concentration of IL-5 [27] and IL-12 [27] and a non-significantly higher concentration of IL-2 [27], IL-6 [27], and IL-13 [27] were found in PMS women compared to controls. Summarizing the results for all interleukins (except IL-10 level in urine in one study [21] and IL-17 serum level in another study [24]), their blood and urine concentrations were higher in PMS cases than in controls.

Among other inflammatory markers, the most commonly studied were tumor necrosis factor α (TNF-α, three papers [21,24,27]), interferon gamma (IFN-γ, two papers [24,27]) and high sensitivity c-reactive protein (hsCRP; two papers [25,27]). A significantly higher serum TNF-α was found in PMS cases than in controls in a medium-quality study by Azizieh et al. [24], whereas the differences between groups were not statistically significant in the other two high-quality studies (including urine [21] and serum samples [27]). The IFN-γ level was higher in PMS cases than in healthy controls in one high-quality [27], but not in another medium-quality study [24]. Moreover, there were no statistically significant results for hsCRP in two high-quality studies included in this systematic review [25,27]. Biomarkers such as granulocyte-macrophage colony-stimulating factor (GMCSF) [27] and Anti-Hsp27 [25] were determined only in single studies, and statistically significant differences in these parameters were not observed between the PMS and control groups.

### 3.2. Oxidative Stress Markers

Among studies included in the systematic review, total oxidative stress indicators, lipid peroxidation, protein oxidation products, and other oxidative stress parameters related to PMS were examined (Table 5). Total oxidative stress was measured only by Incebiyk et al. [22] using total oxidant status (TOS) and oxidative stress index (OSI) without statistically significant differences between the PMS and control groups. Three studies reported results on lipid peroxidation [18,19,22] and two on protein oxidation [19,23] products. The majority of them showed no significant differences between PMS cases and control groups, except a high-quality study by Duvan et al. [19], in which a higher level of lipid hydroperoxide (LHP; an early marker of the oxidation chain of lipids) was found in PMS women compared to controls.

From the other oxidative stress markers, only four parameters were determined in the studies included in this review [18,19,22]. Among studied markers, i.e., free sulfhydryl groups [22] (-SH), total thiol [19] (T-SH), nitric oxide [18] (NO), and adrenomedullin [18] (AM), only the AM plasma concentration was significantly higher in the PMS versus the control group in a high-quality study [18].

### 3.3. Antioxidative Status Parameters

#### 3.3.1. Nonenzymatic Antioxidant Parameters

Four studies included in the review examined total antioxidant capacity (TAC) in blood in PMS cases and controls [19,20,22,23] (Table 6). A statistically significantly lower TAC was found in the PMS women than in the control group in two high-quality [19,20], but not in two medium-quality studies [22,23].

Five studies comparing specific blood levels of vitamins and minerals in PMS cases and healthy controls were analyzed [20,24,25,26,28] (Table 6). Only in individual study, vitamin A and vitamin E levels were determined [25], and women suffering from PMS have characterized statistically significantly lower concentrations of vitamin A but not vitamin E. Vitamin D concentration, which is considered to play an essential role in the anti- and pro-oxidant regulations [29,30], was examined in two medium-quality studies [24,28]; however, only the results of one of them demonstrated a significantly lower level of vitamin D in PMS cases versus the control group [28]. Zinc levels were determined in three studies [20,26,28], but statistically significant results were obtained only in one high-quality study by Fathizadeh et al. [20], where PMS cases had lower serum zinc levels than controls. Copper serum level was measured exclusively by Bahrami et al. [26]. Although PMS cases versus controls had two times lower copper levels in serum (9.2 vs. 18.4 μmol/L), the copper to zinc ratio was relatively similar in both groups [26].

#### 3.3.2. Enzymatic Antioxidants

We did not identify studies that included a variety of antioxidant enzyme markers, which met the review eligible criteria. Only in one paper superoxide dismutase (SOD) activity was measured and results for both groups were similar [26].

## 4. Discussion

To the best of our knowledge, this is the first systematic review to clarify potential associations of inflammation, oxidative stress, and antioxidants status with PMS. Limited evidence suggests that women with PMS may have higher levels of some inflammatory parameters and lower antioxidants status. Evidence regarding oxidative stress is insufficient to formulate a clear statement at this time.

In the studies included in this systematic review, levels of some mediators considered typical for chronic inflammation such as hsCRP [25,27], TNF-α [21,24,27], and IL-1 family [21,24,27], were higher in PMS cases compared to controls, but probably due to the small number of participants in some studies, the differences between groups did not reach statistical significance. Moreover, results of a longitudinal cohort study (2939 midlife women) indicate that the most common premenstrual symptoms, such as mood, abdominal cramps/back pain, appetite cravings, and breast pain/weight gain/bloating, were statistically significantly positively associated with hsCRP level [31]. Furthermore, Puder et al. [32] showed that hsCRP levels change across the whole menstrual cycle in PMS cases simultaneously with psychological and physical symptoms, which indicates the possible role of low-grade inflammation in the pathogenesis of PMS.

Only a few studies regarding oxidative stress in PMS are available; wherein in this review, four studies were included with inconsistent results [18,19,22,23]. Therefore, based on the results, the role of oxidative stress in PMS occurrence and severity is impossible to establish. However, the results of other studies, not included mainly due to methodological issues in the review, may slightly suggest a potential role of this process. For example, in a recently published study, in women suffering from menstrual cycle-associated syndrome (which includes PMS) versus healthy controls, a statistically significantly higher lipid peroxidation, but not advanced oxidation protein products and free sulfhydryl groups, was found [33]. In another study, PMS women had a statistically significantly elevated DNA damage marker (urine 8-hydroxy-2-deoxyguanosine) compared to controls [34]. A higher level of oxidative stress occurs also in another menstrual cycle abnormality—primary dysmenorrhea, which was especially evident for lipid peroxidation markers [35].

Regarding vitamins involved in anti-inflammatory and antioxidant actions, vitamin D was assessed in two independent studies, where lower levels in PMS cases were found [24,28]. Vitamin D may play an important role in PMS via anti-inflammatory and immunomodulatory actions and take part in the calcium metabolism, where any disturbance may lead to emotional and physical symptoms typical for premenstrual syndrome [36]. Many interventional studies were conducted using vitamin D supplements to reduce premenstrual symptoms; however, with inconclusive results [37,38,39,40]. The most pronounced effects were obtained when supplementation was ordered in women with vitamin D hypovitaminosis [37]. In a cross-sectional study (not included in this review due to the lack of a control group), women with inadequate vitamin D status had an increased risk of experiencing some premenstrual symptoms such as mild confusion and severe fatigue or anxiety [41]. Results of two independent systematic reviews of the literature [42,43] revealed that vitamin D may play an important role in PMS occurrence; however, more randomized controlled trials are needed. Regarding other vitamins with antioxidant properties (vitamin A and E), the evidence was limited to a single study [25] and it is impossible to formulate any conclusions in this matter. To the best of our knowledge, there were no studies assessing vitamin C blood level in women with PMS.

Zinc status was determined in three studies [20,26,28] included in this systematic review; however, only in one study, its level was significantly lower in the PMS women compared to the control group [20]. Serum zinc levels vary during the menstrual cycle. In two studies not included in this review due to methodological issues, women with PMS had lower zinc levels than healthy controls, especially in the luteal phase [44,45]. There was found that long-term zinc deficiency can lead to reduced zinc concentration in the hippocampus [46]. This induces disturbance in the glucocorticoid’s production, which may be reflected in some neuropsychological symptoms common in PMS such as irritability, emotional instability, or depression [46,47].

In summary, it can be suggested that a reduced antioxidant capacity in PMS cases may be probable; however, the role of increased oxidative stress remains uncertain. Due to the cross-sectional or case–control design of the included studies, it cannot be judged whether the potential imbalance of oxidant–antioxidant status is the reason for occurring symptoms or a consequence. With a limited number of studies and small sample sizes, it is impossible to verify the hypothesis that oxidant–antioxidant balance plays a significant role in PMS occurrence.

### 4.1. Potential Mechanism

#### 4.1.1. Inflammation

**Figure 2:**
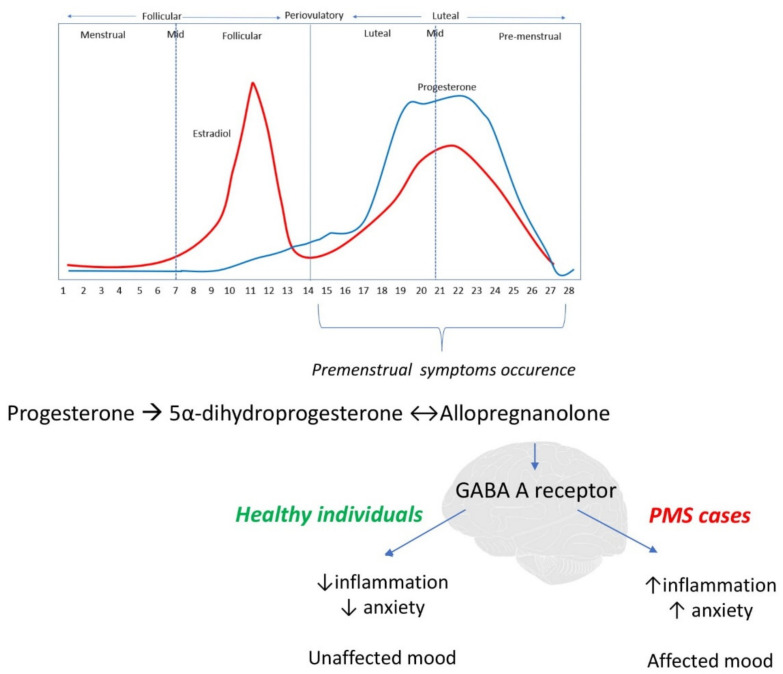
Inflammation in the development of premenstrual symptoms—potential mechanism. Premenstrual symptoms occur in the luteal phase of the menstrual cycle, after high exposure to progesterone and its metabolite—allopregnanolone, which is an agonist of γ-aminobutyric acid A receptor (GABA A receptor). In healthy individuals, allopregnanolone via the GABA A receptor has anxiolytic and sedative effects, which result in unaffected mood, while in PMS cases, it acts in the opposite way which is thought to play a role in premenstrual symptom development.

Despite extensive research, PMS’s etiology remains unknown [1,8]. It is known that an inadequate response to inflammation is one of the major factors in the pathogenesis of multiple chronic diseases [48,49], including the development and course of psychiatric issues [50,51]. Considering that PMS is a combination of mental and physical symptoms, we hypothesized that inadequate response to inflammation might take part in PMS occurrence. The immune system is involved in many actions of female reproductive function, i.e., follicular recruitment, ovulation, and endometrial repair [12], and all women of childbearing age experience changes of inflammation across the menstrual cycle [52]. A sequence of local and inter-cellular inflammatory interactions in the endometrium occurs in the late luteal phase due to the decline in estradiol and progesterone levels, which have anti-inflammatory properties [33]. The magnitude of cyclic changes in inflammatory markers is not the same in all women, and some researchers indicate an essential role of chronic inflammation in PMS etiology [13,14]. One of the proposed hypotheses that seems crucial to fully understand PMS and inflammation’s potential connection is altered sensitivity to physiological hormonal fluctuations. Premenstrual symptoms occur during the luteal phase of the menstrual cycle (7–14 days before the onset of menstruation) [7] along with rapid changes of progesterone and estradiol levels, which rise in the first part of the luteal phase and then fall rapidly [53], as presented in Figure 2. Generally, sex steroid hormone levels are the same in PMS cases and healthy controls; however, it is suspected that women with PMS are characterized by altered sensitivity to physiological hormonal fluctuations, especially estrogen and progesterone, due to a genetic susceptibility [14]. As sex steroids and their derivates (such as allopregnanolone—progesterone metabolite) pass the blood–brain barrier easily, and their receptors are present in essential brain regions such as the amygdala or hypothalamus [1], it is plausible that they may impact the PMS symptoms. Progesterone metabolite—allopregnanolone—is an agonist of γ-aminobutyric acid A receptor (GABA A) with different actions depending on its concentration. In high concentration, allopregnanolone has anxiolytic and sedative effects, while at a lower concentration, it may cause negative mood and depression [8]. GABA A receptors become less sensitive to allopregnanolone after exposure to its high concentrations (as in the first half of the luteal phase), resulting in increased premenstrual tension [8]. The link between allopregnanolone and inflammation is quite complex: results of animal studies indicate that agonists of GABA A receptors attenuate the impact of inflammation, while the inhibition of GABA A receptor activity enhances pro-inflammatory effects [14]. Even though allopregnanolone is meant to reduce inflammation, decrease anxiety and improve mood, in women with PMS it has an opposite effect, presumably due to a change in GABA A receptor setup in the luteal phase, which still requires confirmation in further research [54] (Figure 2).

#### 4.1.2. Oxidative Stress

**Figure 3:**
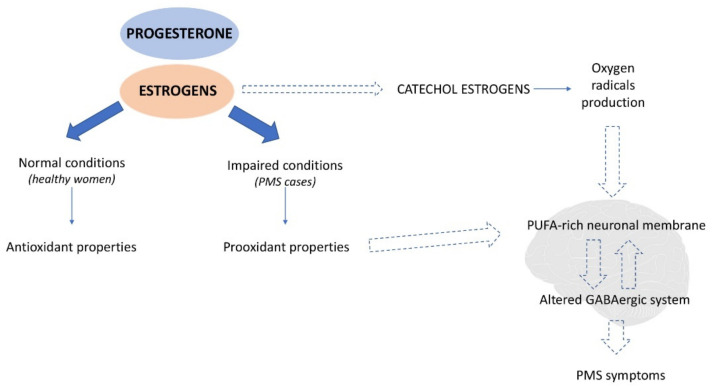
Potential mechanism of the role of oxidative stress in premenstrual symptom development. In healthy women, progesterone and estrogens act as antioxidants; however, in PMS cases, it is suspected that due to improper, increased prooxidant activity, they may cause oxidative damage to the polyunsaturated fatty acid-rich neuronal membrane. Therefore, they alter the GABAergic system, of which dysfunction possibly leads to the PMS symptom development. Another source of oxidative damage in the neuronal membrane might be catechol estrogens (products of estrogens conversion), which produce oxygen radicals.

Progestins and estrogens have anti-inflammatory actions as well as they may act as potential antioxidants [55]. However, similarly to other antioxidants in excessive doses, activities may have a pro-oxidative effect and lead to the disturbance of the oxidative balance towards oxidative stress [56]. As mentioned before, women with PMS, despite having similar hormone levels compared to healthy women, may be more sensitive to its fluctuations; therefore, they can be exposed to excessive estrogen activity in the luteal phase, which stimulates the pro-oxidant nature of estrogens (Figure 3). Additionally, the products of estrogen’s conversion—catechol estrogens, can produce oxygen radicals [57], which may alter the GABAergic system (via polyunsaturated fatty acid-rich neuronal membrane damage) and its dysfunction possibly leads to the PMS symptoms’ development. However, it cannot be concluded whether the imbalance of oxidant–antioxidant status is one of the causes of the altered GABAergic system in PMS cases or a consequence of the hypersensitivity to hormonal fluctuation and exposition to excessive estrogen activity [19].

### 4.2. Strengths and Limitations

To the best of our knowledge, this is the first systematic review to clarify potential associations of inflammation, oxidative stress, and antioxidants status with PMS. This review’s strength is primarily strict compliance with instruction for performing a systemic review recommended by PRISMA [58]. Two independent researchers searched databases and pre-selected publications for review; the studies’ quality assessment was also made independently by two researchers. We included only studies of high and medium quality according to previously established and widely acclaimed criteria [16,17]. We made every effort to include studies conducted on different populations, which was partially limited by publications’ availability, i.e., 36% of the studies included were conducted in Iran. In the systematic review, we included studies evaluating various markers of inflammation, oxidative stress, and antioxidant status to provide readers with a complete picture, as different markers may point to different stages of damage caused by inflammation or oxidative stress.

This paper also has several limitations. Although the quality assessment of the studies included in the review showed that nearly 64% of them were of high quality and none were considered low quality, the sample sizes in particular studies were relatively small, leading to difficulties in reaching statistically significant differences. There was also a high heterogeneity in the study designs, and the diagnosis of PMS was not always made with a validated method. Moreover, adjustment for potential confounders was made only in one study [27], although it can influence the results. Only in one study [22], women taking antioxidant medication and supplements were excluded from the study, in six studies [20,23,24,25,26,28] the authors excluded women taking any kind of medication/supplementation in the previous 6 months, while in the rest of the studies this factor was not taken into consideration, which could have contributed to the lack of significant results in these studies. The number of case–control studies included in the review was limited to six publications [18,19,20,21,22,23], and some included a small number of markers [18,20]; therefore, we have also included cross-sectional studies [24,25,26,27,28]. Despite this, due to the data’s character and the small number of studies that analyzed the same parameters, we could not conduct a meta-analysis.

### 4.3. Implications for Further Research

Due to the fact that PMS is a common problem of unspecified etiology that can significantly lower the quality of women’s lives, it is important to point out and research possible mechanisms that may lead to symptoms. Therefore, it is necessary to establish the role of inflammation, oxidative stress and antioxidants in the pathogenesis of PMS. There is a need to conduct more homogeneous research with a strict diagnosis of PMS made using a validated method, strict inclusion and exclusion criteria, including supplement intake, and adjust results for possible confounders. Moreover, it is relevant to arrange a psychiatrist consultation for the potential study participants to rule out undiagnosed illnesses, which may have symptoms similar to those typical in PMS. It is crucial to investigate the meaning of inflammation, oxidative stress, and antioxidant status in PMS development because of the potential clinical implications for treatment and prevention by raising awareness among women about factors that may potentially reduce PMS. To these factors, among others we include a healthy diet, reducing overweight and obesity when occurring, avoiding a sedentary lifestyle, and quitting smoking.

## 5. Conclusions

Limited evidence indicates that women with PMS may have higher levels of some inflammatory parameters and suggests lower antioxidants status. Data on the contribution of oxidative stress in women with PMS are insufficient to formulate conclusions. Obtained results are of great importance for public health due to the common prevalence of premenstrual syndrome and its negative impact on life quality. Based on the present findings, and with all the limitations described above, well-designed research should be considered in the future to develop firm conclusions on the associations between inflammation, oxidative stress, and antioxidant status versus PMS.

## Figures and Tables

**Table 1:** Quality assessment of studies included in the systematic review using a modified Newcastle–Ottawa Quality Assessment Scale for case–control studies [16] and cross-sectional studies [17].

Case–Control Studies					
Authors	Selection (Max. 4 Stars)	Comparability (Max. 2 Stars)	Exposure (Max. 3 Stars)	Total Points (Max. 9)	Quality
Balat et al. [18]	3	2	2	7	High
Duvan et al. [19]	3	2	2	7	High
Fathizadeh et al. [20]	3	2	2	7	High
Foster et al. [21]	3	2	2	7	High
Incebiyik et al. [22]	1	2	1	4	Medium
Tuladhar and Rao [23]	2	2	2	6	Medium
**Cross-Sectional Studies**					
**Authors**	**Selection** **(Max. 5 Stars)**	**Comparability** **(Max. 2 Stars)**	**Outcome** **(Max. 3 Stars)**	**Sum** **(Max. 10)**	**Quality**
Azizieh et al. [24]	1	2	2	5	Medium
Bahrami et al. [25]	3	2	3	8	High
Bahrami et al. [26]	2	2	3	7	High
Bertone-Johnson et al. [27]	2	2	3	7	High
Fatemi et al. [28]	2	0	3	5	Medium

Case–control studies (range of total points 0–9): 0 to 3 points—low-quality study, 4 to 6 points—medium-quality study, and 7 to 9 points—high-quality study. Cross-sectional studies (range of total points 0–10): 0 to 3 points—low-quality study, 4 to 6 points—medium-quality study, and 7 to 10 points—high-quality study.

**Table 2:** Characteristics of the studies included in the systematic review.

Authors	Location	Cases n	Case Definition	Controls n	Participants Characteristics	Age Cases Mean ± SD (Range), Years	Age Controls Mean ± SD (Range), Years	Biomarkers of Interest	Adjustment
Case–control studies									
Balat et al. [18]	Turkey	11	ACOG, DSRS	10	non-pregnant, not under treatment for PMS, no history of psychiatric disorders, not taking oral contraceptives	34.2 ± 3.5 (28–37)	32.9 ± 3.1 (28–37)	MDA, NO, AM	no
Duvan et al. [19]	Turkey	20	DSRS	21	regular menses for at least six previous cycles, non-pregnant, non-breastfeeding, not under PMS treatment, no history of psychiatric disorders, thyroid and other endocrine disorders, not taking oral contraceptive pills	29.23 ± 4.79 (22–39)	28.05 ± 4.66 (22–39)	LHP, MDA, PC, T-SH, TAC	no
Fathizadeh et al. [20]	Iran	23	DSRS	25	medical students from dormitories, no history of chronic disease, no nutritional supplements or medication intake *	24.17 ± 0.55 (21–31)	23.64 ± 0.60 (21–31)	TAC, zinc	no
Foster et al. [21]	Brazil	31	DSRS	21	non-pregnant, regularly attending training sessions, with regular menstrual cycles, no fractures, no severe ligament injuries, no presented genetic or acquired kidney disease; no hormonal contraceptive or antidepressants, anxiolytics, diuretics, steroids or illegal drugs	18.7 ± 3.99 (N/A)	20.68 ± 3.70 (N/A)	IL-1β, IL-6, IL-8, IL-10, TNF-α	no
Incebiyik et al. [22]	Turkey	40	ACOG, DSRS	40	non-pregnant, non-breastfeeding, with regular menstrual cycles, no chronic illnesses, such as thyroid or metabolic diseases; no psychiatric disorders, such as psychosis or bipolar disorder; no hormonal contraceptives, no serotonin reuptake inhibitors or antidepressants; no antioxidant medication, no previous hysterectomy or ovarian surgery, no smokers	28.93 ± 7.16 (18–45)	29.45 ± 5.10 (18–45)	TAC (TAS), TOS, OSI, LHP (LOOH), -SH	no
Tuladhar and Rao [23]	India	74	COPE	80	regular menstrual cycles with no other illness and were not on any medications, no history of polycystic ovarian disease, smoking, alcohol consumption, drug abuse, insulin resistance and use of contraceptive pills	23 ** (20–24)	24 ** (20–24)	TAC (FRAP), PPT, PC	no
Cross-sectional studies									
Azizieh et al. [24]	Kuwait	94	self-assessment	23	Kuwaiti citizens only; non-pregnant, non-breastfeeding, free from infections/chronic diseases, and not taking any medications or vitamin/mineral supplements/injections for the previous 6 months	28.5 (19–47)	25 (19–47)	IL-1β, IL-8, IL-17, IL-4, TNF-α, IFN-γ, vitamin D	no
Bahrami et al. [25]	Iran	134	COPE	148	without any autoimmune abnormalities, carcinoma, metabolic or cardiovascular disorders (CVD), liver or renal failure, periodontal disease, and endocrinopathy diagnosed by physicians or self-reported; no anti-inflammatory, antidepressant, vitamins supplement consumption and hormone therapy over the past year	14.6 ± 1.4 (12–18)	14.6 ± 1.7 (12–18)	Anti-Hsp27, hsCRP, vitamin A, vitamin E	no
Bahrami et al. [26]	Iran	67	COPE	74	no acute or chronic physical/psychological disease even without drug consumption; no mineral supplementation during the six months before recruitment	(12–18)	(12–18)	zinc, copper, zinc/copper ratio, SOD	no
Bertone-Johnson et al. [27]	USA	37	modified COPE	67	currently menstruating women; not reporting a history of high blood pressure or elevated cholesterol, kidney or liver disease, bone disease such as osteomalacia, digestive disorders, rheumatologic disease, multiple sclerosis, thyroid disease, hyperparathyroidism, cancer, type 1 or type 2 diabetes or polycystic ovaries; not taking corticosteroids, anabolic steroids, anticonvulsants, cimetidine or propranolol.	21.6 ± 3.1 (18–30)	21.1 ± 2.7 (18–30)	IL-1β, IL-2, IL-4, IL-5, IL-6, IL-7, IL-8, IL-10, IL-12, IL-13, TNF-α, GMCSF, IFN-γ, hsCRP	age, BMI, smoking status
Fatemi et al. [28]	Iran	115	PSST	163	no endocrine disorders, no vitamin and mineral supplements, no corticosteroid antidepressants, no oral contraceptive pill	N/A (19–21)	N/A (19–21)	zinc, vitamin D	no

* Information obtained via direct contact with the author; ** Results presented as median (range). Abbreviations: ACOG—American College of Obstetrics and Gynecology, AM—adrenomedullin, BMI—body mass index, COPE—calendar of premenstrual experiences, DSRS—daily symptom rating scale, GMCSF—granulocyte macrophage colony stimulating factor, GSH—glutathione, hsCRP—high sensitivity C-reactive protein, IFNγ—interferon gamma, IL—interleukin, LHP (LOOH) —lipid hydroperoxide, MDA—malondialdehyde, N/A—not available, NO—nitric oxide, OSI—oxidative stress index, PC—protein carbonyl, PPT—plasma protein thiol, PSST—premenstrual symptoms screening tool, -SH—free sulfhydryl groups, SOD—superoxide dismutase, TAC (TAS/FRAP)—total antioxidant capacity (total antioxidant status/ferric reducing antioxidant power of plasma), TNF-α—tumor necrosis factor α, TOS—total oxidant status, T-SH—total thiol, PMS—premenstrual syndrome.

**Table 3:** Summary of the biomarkers included in the studies included in the systematic review.

Inflammatory Parameters	Oxidative Stress Parameters	Antioxidative Status Parameters
Interleukins	Total	Total
IL-1β [21,24,27]	TOS [22]	TAC (TAS/FRAP) [19,20,22,23]
IL-2 [27]	OSI [22]	Specific vitamins/minerals
IL-4 [24,27]	Lipid peroxidation and protein oxidation	vitamin A [25]
IL-5 [27]	MDA [18,19]	vitamin D [24,28]
IL-6 [27]	LHP (LOOH) [19,22]	vitamin E [25]
IL-7 [21,27]	PC [19,23]	cooper [26]
IL-8 [21,24,27]	PPT [23]	zinc [20,26,28]
IL-10 [21,27]	Others	zinc/cooper ratio [26]
IL-12 [27]	-SH [22]	Enzymes
IL-13 [27]	T-SH [19]	SOD [26]
IL-17 [24]	NO [18]	
Others	AM [18]	
TNF-α [21,24,27]		
IFN-γ [24,27]		
GMCSF [27]		
hsCRP [25,27]		
Anti-Hsp27 [25]		

Abbreviations: AM—adrenomedullin, GMCSF—granulocyte-macrophage colony-stimulating factor, hsCRP—high sensitivity c-reactive protein, IFNγ—interferon gamma, IL—interleukin, LHP (LOOH) —lipid hydroperoxide, MDA—malondialdehyde, NO—nitric oxide, OSI—oxidative stress index, PC—protein carbonyl, PPT—plasma protein thiol, -SH—free sulfhydryl groups, SOD—superoxide dismutase, TAC (TAS/FRAP)—total antioxidant capacity (total antioxidant status/ferric reducing antioxidant power of plasma), TNF-α—tumor necrosis factor α, TOS—total oxidant status, T-SH—total thiol.

**Table 4:** Inflammatory markers in serum and urine of women with PMS and controls.

Authors	Biological Sample	Results Presentation Method (Unit)	No. of Cases	No. of Controls	Results PMS Cases	Results Controls	*p*-Value
INTERLEUKINS							
IL-1β							
Azizieh et al. [24]	Serum	Median (pg/mL)	94	23	0.3	0.2	0.32
Bertone-Johnson et al. [27]	Serum	Geometric mean (pg/mL)	37	67	0.72	0.28	0.27
Foster et al. [21]	Urine	Square mean root ± SD (pg/mL)	31	21	1.12 ±0.23	0.90 ± 0.25	0.01
IL-2							
Bertone-Johnson et al. [27]	Serum	Geometric mean (pg/mL)	37	67	1.94	1.09	0.09
IL-4							
Azizieh et al. [24]	Serum	Median (pg/mL)	94	23	163.9	35.2	0.18
Bertone-Johnson et al. [27]	Serum	Geometric mean (pg/mL)	37	67	8.01	4.16	0.01
IL-5							
Bertone-Johnson et al. [27]	Serum	Geometric mean (pg/mL)	37	67	0.33	0.20	0.05
IL-6							
Bertone-Johnson et al. [27]	Serum	Geometric mean (pg/mL)	37	67	2.68	1.88	0.27
IL-7							
Bertone-Johnson et al. [27]	Serum	Geometric mean (pg/mL)	37	67	2.11	1.20	0.09
Foster et al. [21]	Urine	Square mean ± SD (pg/mL)	31	21	9.48 ± 1.68	6.45 ± 1.01	0.001
IL-8							
Azizieh et al. [24]	Serum	Median (pg/mL)	94	23	14.5	10.7	0.009
Bertone-Johnson et al. [27]	Serum	Geometric mean (pg/mL)	37	67	2.26	1.72	0.20
Foster et al. [21]	Urine	Square mean ± SD (pg/mL)	31	21	1.26 ± 0.48	0.81 ± 0.10	0.04
IL-10							
Bertone-Johnson et al. [27]	Serum	Geometric mean (pg/mL)	37	67	17.15	9.19	0.03
Foster et al. [21]	Urine	Square mean ± SD (pg/mL)	31	21	0.98 ± 0.12	0.97 ± 0.09	0.74
IL-12							
Bertone-Johnson et al. [27]	Serum	Geometric mean (pg/mL)	37	67	5.57	2.06	0.04
IL-13							
Bertone-Johnson et al. [27]	Serum	Geometric mean (pg/mL)	37	67	2.35	1.23	0.08
IL-17							
Azizieh et al. [24]	Serum	Median (pg/mL)	94	23	4.0	4.3	0.47
OTHERS							
TNF-α							
Azizieh et al. [24]	Serum	Median (pg/mL)	94	23	10.6	7.6	0.002
Bertone-Johnson et al. [27]	Serum	Geometric mean (pg/mL)	37	67	4.78	4.35	0.59
Foster et al. [21]	Urine	Square mean ± SD (pg/mL)	31	21	1.23 ± 0.36	1.15 ± 0.23	0.58
IFN-γ							
Azizieh et al. [24]	Serum	Median (pg/mL)	94	23	11.0	10.7	0.97
Bertone-Johnson et al. [27]	Serum	Geometric mean (pg/mL)	37	67	3.59	1.39	0.01
hsCRP							
Bahrami et al. [25]	Serum	Median (IR) (mg/L)	134	148	0.98 (0.64–1.72)	0.82 (0.41–1.61)	>0.05
Bertone-Johnson et al. [27]	Serum	Geometric mean (mg/L)	28	41	1.36	0.91	0.30
GMCSF							
Bertone-Johnson et al. [27]	Serum	Geometric mean (pg/mL)	37	67	8.42	5.41	0.23
Anti-Hsp27							
Bahrami et al. [25]	Serum	Mean ± SD * (N/A)	134	148	0.26 ± 0.17	0.29 ± 0.24	>0.05

* All such terms presented as arithmetic mean, Abbreviations: GMCSF—granulocyte-macrophage colony-stimulating factor, hsCRP—high sensitivity C-reactive protein, IFN-γ—interferon gamma, IL—interleukin, IR—interquartile range, N/A—not available, SD—standard deviation, TNF-α—tumor necrosis factor α.

**Table 5:** Oxidative stress parameters in blood of women with PMS and controls.

Authors	Biological Sample	Results Presentation Method (Unit)	No. of Cases	No. of Controls	Results PMS Cases	Results Controls	*p*-Value
TOTAL OXIDATIVE STATUS							
TOS							
Incebiyik et al. [22]	Serum	Mean ± SD * (μmol H_2_O_2_ equivalent/L)	40	40	46.78 ± 23.32	41.89 ± 18.64	0.304
OSI							
Incebiyik et al. [22]	Serum	Mean ± SD (mmol trolox/L)	40	40	5.10 ± 2.87	4.44 ± 2.12	0.243
LIPID PEROXIDATION AND PROTEIN OXIDATION							
MDA							
Balat et al. [18]	Plasma	Mean ± SD (N/A)	11	10	1.4 ± 0.1	1.6 ± 0.1	>0.05
Duvan et al. [19]	Plasma	Mean ± SD (nmol/mL)	20	21	4.46 ± 0.54	4.50 ± 0.46	0.78
LHP (LOOH)							
Duvan et al. [19]	Plasma	Mean ± SD (nmol/mL)	20	21	0.28 ± 0.08	0.20 ± 0.04	0.01
Incebiyik et al. [22]	Serum	Mean ± SD (mmol H_2_O_2_ equivalent/l)	40	40	23.21 ± 13.80	20.43 ± 10.90	0.321
PC							
Duvan et al. [19]	Serum	Mean ± SD (nmol/mL)	20	21	13.9 ± 1.38	13.4 ± 1.45	0.18
Tuladhar and Rao [23]	Plasma	Median (IR) (nmol/mg protein)	74	80	0.90 (0.68–1.20)	0.84 (0.65–1.12)	>0.05
PPT							
Tuladhar and Rao [23]	Plasma	Median (IR) (µmol)	74	80	410 (371–449)	410 (371–468)	>0.05
OTHERS							
-SH							
Incebiyik et al. [22]	Serum	Mean ± SD (mmol/L)	40	40	0.46 ± 0.09	0.43 ± 0.06	0.064
T-SH							
Duvan et al. [19]	Serum	Mean ± SD (nmol/L)	20	21	0.39 ± 0.07	0.37 ± 0.05	0.59
NO							
Balat et al. [18]	Plasma	Mean ± SD (N/A)	11	10	42 ± 2.4	43 ± 2.4	>0.05
AM							
Balat et al. [18]	Plasma	Mean ± SD (N/A)	11	10	37 ± 2.2	26 ± 1.4	<0.05

* All such terms presented as arithmetic mean, Abbreviations: AM—adrenomedullin, IR—interquartile range, LHP (LOOH)—lipid hydroperoxide, MDA—malondialdehyde, N/A—not available, NO—nitric oxide, OSI—oxidative stress index, PC—protein carbonyl, PPT—plasma protein thiol, -SH—free sulfhydryl groups, TOS—total oxidant status, T-SH—total thiol.

**Table 6:** Antioxidant status parameters in the blood of women with PMS and controls.

Authors	Biological Sample	Results Presentation Method (Unit)	No. of Cases	No. of Controls	Results PMS Cases	Results Controls	*p*-Value
*Nonenzymatic Antioxidant Parameters*							
Total Antioxidant Status Parameters							
TAC (TAS/FRAP)							
Duvan et al. [19]	Plasma	Mean ± SD * (mmol/L)	20	21	0.55 ± 0.22	0.73 ± 0.09	0.01
Fathizadeh et al. [20]	Serum	Mean ± SD (mmol/L)	23	25	0.81 ± 0.041	1.075 ± 0.06	<0.01
Incebiyik et al. [22]	Serum	Mean ± SD (mmol Trolox equivalent/L)	40	40	0.96 ± 0.19	0.96 ± 0.15	0.982
Tuladhar and Rao [23]	Plasma	Median (IR) (µmol/L)	74	80	955 (775–1110)	1020 (860–1175)	>0.05
Specific Vitamins And Minerals							
Vitamin A							
Bahrami et al. [25]	Serum	Median (IR) (μmol/L)	134	148	1.12 (0.26–9.30)	7.2 (2.66–19.27)	0.007
Vitamin D							
Azizieh et al. [24]	Serum	Median (nmol/L)	94	23	17.0	14.0	0.30
Fatemi et al. [28]	Serum	Mean ± SD (ng/mL)	115	163	22.44 ± 13.91	26.58 ± 6.74	0.043
Vitamin E							
Bahrami et al. [25]	Serum	Median (IR) (μmol/L)	134	148	3.75 (2.87–5.80)	4.0 (2.69–5.80)	>0.05
Cooper							
Bahrami et al. [26]	Serum	Mean ± SD (μmol/L)	67	74	9.2 ± 7.6	18.4 ± 9.0	NCD **
Zinc							
Bahrami et al. [26]	Serum	Mean ± SD (μmol/L)	67	74	14.3 ± 3.1	14.3 ± 14.3	NCD **
Fatemi et al. [28]	Serum	Mean ± SD (μg/dl)	115	163	83.49 ± 11.58	84.41 ± 11.80	0.521
Fathizadeh et al. [20]	Serum	Mean ± SD (μg/dl)	23	25	108.20 ± 3.73	153.8 ± 18.77	0.026
Zinc to cooper ratio							
Bahrami et al. [26]	Serum	Mean ± SD	67	74	0.8 ± 0.4	1.0 ± 7.0	NCD **
*Enzymes*							
SOD							
Bahrami et al. [26]	Serum	Mean ± SD (U/mL)	67	74	0.05 ± 0.02	0.05 ± 0.02	NCD **

* All such terms presented as arithmetic mean; ** Not compared directly (NCD)—due to more groups in the study, the PMS and the control group were not compered directly. Abbreviations: IR—interquartile range, SOD—superoxide dismutase, TAC (TAS/FRAP)—total antioxidant capacity.

## Supplementary Materials

The following are available online at https://www.mdpi.com/article/10.3390/antiox10040604/s1.

## References

[1] Yonkers K.A., Simoni M.K. (2018). Premenstrual Disorders. Am. J. Obstet. Gynecol..

[2] Hofmeister S., Bodden S. (2016). Premenstrual Syndrome and Premenstrual Dysphoric Disorder. Am. Fam. Physician.

[3] Direkvand-Moghadam A., Sayehmiri K., Delpisheh A., Kaikhavandi S. (2014). Epidemiology of Premenstrual Syndrome (PMS)—A Systematic Review and Meta-Analysis Study. J. Clin. Diagn. Res. JCDR.

[4] O’Brien S., Rapkin A., Dennerstein L., Nevatte T. (2011). Diagnosis and Management of Premenstrual Disorders. BMJ.

[5] Schmidt P.J., Martinez P.E., Nieman L.K., Koziol D.E., Thompson K.D., Schenkel L., Wakim P.G., Rubinow D.R. (2017). Premenstrual Dysphoric Disorder Symptoms Following Ovarian Suppression: Triggered by Change in Ovarian Steroid Levels but Not Continuous Stable Levels. Am. J. Psychiatry.

[6] Spinelli M.G. (2004). Depression and Hormone Therapy. Clin. Obstet. Gynecol..

[7] Appleton S.M. (2018). Premenstrual Syndrome: Evidence-Based Evaluation and Treatment. Clin. Obstet. Gynecol..

[8] Walsh S., Ismaili E., Naheed B., O’Brien S. (2015). Diagnosis, Pathophysiology and Management of Premenstrual Syndrome. Obstet. Gynaecol..

[9] Hussain T., Tan B., Yin Y., Blachier F., Tossou M.C.B., Rahu N. (2016). Oxidative Stress and Inflammation: What Polyphenols Can Do for Us?. Oxid. Med. Cell. Longev..

[10] Birben E., Sahiner U.M., Sackesen C., Erzurum S., Kalayci O. (2012). Oxidative Stress and Antioxidant Defense. World Allergy Organ. J..

[11] Dandekar A., Mendez R., Zhang K. (2015). Cross Talk between ER Stress, Oxidative Stress, and Inflammation in Health and Disease. Methods Mol. Biol..

[12] Mattina G.F., van Lieshout R.J., Steiner M. (2019). Inflammation, Depression and Cardiovascular Disease in Women: The Role of the Immune System across Critical Reproductive Events. Ther. Adv. Cardiovasc. Dis..

[13] Bertone-Johnson E.R. (2016). Chronic Inflammation and Premenstrual Syndrome: A Missing Link Found?. J. Womens Health.

[14] Bannister E. (2019). There Is Increasing Evidence to Suggest That Brain Inflammation Could Play a Key Role in the Aetiology of Psychiatric Illness. Could Inflammation Be a Cause of the Premenstrual Syndromes PMS and PMDD?. Post Reprod. Health.

[15] O’Brien P.M.S., Bäckström T., Brown C., Dennerstein L., Endicott J., Epperson C.N., Eriksson E., Freeman E., Halbreich U., Ismail K.M.K. (2011). Towards a Consensus on Diagnostic Criteria, Measurement and Trial Design of the Premenstrual Disorders: The ISPMD Montreal Consensus. Arch. Womens Ment. Health.

[16] Wells G.A., Shea B., O’Connell D., Peterson J., Welch V., Losos M., Tugwell P. The Newcastle-Ottawa Scale (NOS) for Assessing the Quality of Nonrandomised Studies in Meta-Analyses. http://www.ohri.ca/programs/clinical_epidemiology/oxford.asp.

[17] Modesti P.A., Reboldi G., Cappuccio F.P., Agyemang C., Remuzzi G., Rapi S., Perruolo E., Parati G. (2016). ESH Working Group on CV Risk in Low Resource Settings Panethnic Differences in Blood Pressure in Europe: A Systematic Review and Meta-Analysis. PLoS ONE.

[18] Balat O., Dikensoy E., Ugur M.G., Atmaca R., Cekmen M., Yurekli M. (2007). Malon Dialdehyde, Nitrite and Adrenomedullin Levels in Patients with Premenstrual Syndrome. Arch. Gynecol. Obstet..

[19] Duvan C.I., Cumaoglu A., Turhan N.O., Karasu C., Kafali H. (2011). Oxidant/Antioxidant Status in Premenstrual Syndrome. Arch. Gynecol. Obstet..

[20] Fathizadeh S., Amani R., Haghighizadeh M.H., Hormozi R. (2016). Comparison of Serum Zinc Concentrations and Body Antioxidant Status between Young Women with Premenstrual Syndrome and Normal Controls: A Case-Control Study. Int. J. Reprod. Biomed. Yazd Iran.

[21] Foster R., Vaisberg M., Bachi A.L.L., dos Santos J.d.M.B., de Paula Vieira R., Luna L.A., Araújo M.P., Parmigiano T.R., Borges F., di-Bella Z.I.K.J. (2019). Premenstrual Syndrome, Inflammatory Status, and Mood States in Soccer Players. Neuroimmunomodulation.

[22] Incebiyik A., Camuzcuoglu A., Hilali N.G., Ulas T., Vural M., Camuzcuoglu H., Aksoy N. (2015). Serum Oxidative Stress, Visfatin and Apelin in Healthy Women and Those with Premenstrual Syndrome. J. Obstet. Gynaecol. J. Inst. Obstet. Gynaecol..

[23] Tuladhar E.T., Rao A. (2010). Plasma Protein Oxidation and Total Antioxidant Power in Premenstrual Syndrome. Asian Pac. J. Trop. Med..

[24] Azizieh F.Y., Alyahya K.O., Dingle K. (2017). Association of Self-Reported Symptoms with Serum Levels of Vitamin D and Multivariate Cytokine Profile in Healthy Women. J. Inflamm. Res..

[25] Bahrami A., Bahrami-Taghanaki H., Khorasanchi Z., Timar A., Jaberi N., Azaryan E., Tayefi M., Ferns G.A., Sadeghnia H.R., Ghayour-Mobarhan M. (2020). Menstrual Problems in Adolescence: Relationship to Serum Vitamins A and E, and Systemic Inflammation. Arch. Gynecol. Obstet..

[26] Bahrami A., Gonoodi K., Khayyatzadeh S.S., Tayefi M., Darroudi S., Bahrami-Taghanaki H., Eslami S., Jaberi N., Ferns G.A., Farahmand K. (2019). The Association of Trace Elements with Premenstrual Syndrome, Dysmenorrhea and Irritable Bowel Syndrome in Adolescents. Eur. J. Obstet. Gynecol. Reprod. Biol..

[27] Bertone-Johnson E.R., Ronnenberg A.G., Houghton S.C., Nobles C., Zagarins S.E., Takashima-Uebelhoer B.B., Faraj J.L., Whitcomb B.W. (2014). Association of Inflammation Markers with Menstrual Symptom Severity and Premenstrual Syndrome in Young Women. Hum. Reprod. Oxf. Engl..

[28] Fatemi M., Allahdadian M., Bahadorani M. (2019). Comparison of Serum Level of Some Trace Elements and Vitamin D between Patients with Premenstrual Syndrome and Normal Controls: A Cross-Sectional Study. Int. J. Reprod. Biomed..

[29] Dzik K.P., Kaczor J.J. (2019). Mechanisms of Vitamin D on Skeletal Muscle Function: Oxidative Stress, Energy Metabolism and Anabolic State. Eur. J. Appl. Physiol..

[30] Pfeffer P.E., Lu H., Mann E.H., Chen Y.-H., Ho T.-R., Cousins D.J., Corrigan C., Kelly F.J., Mudway I.S., Hawrylowicz C.M. (2018). Effects of Vitamin D on Inflammatory and Oxidative Stress Responses of Human Bronchial Epithelial Cells Exposed to Particulate Matter. PLoS ONE.

[31] Gold E.B., Wells C., Rasor M.O. (2016). The Association of Inflammation with Premenstrual Symptoms. J. Womens Health.

[32] Puder J.J., Blum C.A., Mueller B., de Geyter C., Dye L., Keller U. (2006). Menstrual Cycle Symptoms Are Associated with Changes in Low-Grade Inflammation. Eur. J. Clin. Investig..

[33] Roomruangwong C., Matsumoto A.K., Michelin A.P., de Oliveira Semeão L., de Lima Pedrão J.V., Moreira E.G., Sirivichayakul S., Carvalho A., Barbosa D.S., Maes M. (2020). The Role of Immune and Oxidative Pathways in Menstrual Cycle Associated Depressive, Physio-Somatic, Breast and Anxiety Symptoms: Modulation by Sex Hormones. J. Psychosom. Res..

[34] Purnawati J., Sinrang A.W., Jusuf E.C., Limoa E., Ahmad M., Usman A.N. (2020). Nutrition, Mental Status and Level of 8-Hydroxy-2-Deoxyguanosine (OHDG) Urine as Predictors of Premenstrual Syndrome (PMS) in Adolescent Girls. Int. J. Curr. Res. Rev..

[35] Szmidt M.K., Granda D., Sicinska E., Kaluza J. (2020). Primary Dysmenorrhea in Relation to Oxidative Stress and Antioxidant Status: A Systematic Review of Case-Control Studies. Antioxidants.

[36] Heidari H., Amani R., Feizi A., Askari G., Kohan S., Tavasoli P. (2019). Vitamin D Supplementation for Premenstrual Syndrome-Related Inflammation and Antioxidant Markers in Students with Vitamin D Deficient: A Randomized Clinical Trial. Sci. Rep..

[37] Tartagni M., Cicinelli M.V., Tartagni M.V., Alrasheed H., Matteo M., Baldini D., de Salvia M., Loverro G., Montagnani M. (2016). Vitamin D Supplementation for Premenstrual Syndrome-Related Mood Disorders in Adolescents with Severe Hypovitaminosis D. J. Pediatr. Adolesc. Gynecol..

[38] Abdollahi R., Abiri B., Sarbakhsh P., Kashanian M., Vafa M. (2019). The Effect of Vitamin D Supplement Consumption on Premenstrual Syndrome in Vitamin D-Deficient Young Girls: A Randomized, Double-Blind, Placebo-Controlled Clinical Trial. Complement. Med. Res..

[39] Bahrami A., Avan A., Sadeghnia H.R., Esmaeili H., Tayefi M., Ghasemi F., Nejati Salehkhani F., Arabpour-Dahoue M., Rastgar-Moghadam A., Ferns G.A. (2018). High Dose Vitamin D Supplementation Can Improve Menstrual Problems, Dysmenorrhea, and Premenstrual Syndrome in Adolescents. Gynecol. Endocrinol..

[40] Dadkhah H., Ebrahimi E., Fathizadeh N. (2016). Evaluating the Effects of Vitamin D and Vitamin E Supplement on Premenstrual Syndrome: A Randomized, Double-Blind, Controlled Trial. Iran. J. Nurs. Midwifery Res..

[41] Jarosz A.C., El-Sohemy A. (2019). Association between Vitamin D Status and Premenstrual Symptoms. J. Acad. Nutr. Diet.

[42] Abdi F., Ozgoli G., Rahnemaie F.S. (2019). A Systematic Review of the Role of Vitamin D and Calcium in Premenstrual Syndrome. Obstet. Gynecol. Sci..

[43] Arab A., Golpour-Hamedani S., Rafie N. (2019). The Association Between Vitamin D and Premenstrual Syndrome: A Systematic Review and Meta-Analysis of Current Literature. J. Am. Coll. Nutr..

[44] Posaci C., Erten O., Uren A., Acar B. (1994). Plasma Copper, Zinc and Magnesium Levels in Patients with Premenstrual Tension Syndrome. Acta Obstet. Gynecol. Scand..

[45] Chuong C.J., Dawson E.B. (1994). Zinc and Copper Levels in Premenstrual Syndrome. Fertil. Steril..

[46] Takeda A., Tamano H. (2009). Insight into Zinc Signaling from Dietary Zinc Deficiency. Brain Res. Rev..

[47] Jafari F., Amani R., Tarrahi M.J. (2020). Effect of Zinc Supplementation on Physical and Psychological Symptoms, Biomarkers of Inflammation, Oxidative Stress, and Brain-Derived Neurotrophic Factor in Young Women with Premenstrual Syndrome: A Randomized, Double-Blind, Placebo-Controlled Trial. Biol. Trace Elem. Res..

[48] Kunnumakkara A.B., Sailo B.L., Banik K., Harsha C., Prasad S., Gupta S.C., Bharti A.C., Aggarwal B.B. (2018). Chronic Diseases, Inflammation, and Spices: How Are They Linked?. J. Transl. Med..

[49] Tsoupras A., Lordan R., Zabetakis I. (2018). Inflammation, Not Cholesterol, Is a Cause of Chronic Disease. Nutrients.

[50] Berk M., Williams L.J., Jacka F.N., O’Neil A., Pasco J.A., Moylan S., Allen N.B., Stuart A.L., Hayley A.C., Byrne M.L. (2013). So Depression Is an Inflammatory Disease, but Where Does the Inflammation Come From?. BMC Med..

[51] Bauer M.E., Teixeira A.L. (2019). Inflammation in Psychiatric Disorders: What Comes First?: Inflammation in Psychiatric Disorders. Ann. N. Y. Acad. Sci..

[52] Lorenz T.K., Demas G.E., Heiman J.R. (2017). Partnered Sexual Activity Moderates Menstrual Cycle–Related Changes in Inflammation Markers in Healthy Women: An Exploratory Observational Study. Fertil. Steril..

[53] Draper C.F., Duisters K., Weger B., Chakrabarti A., Harms A.C., Brennan L., Hankemeier T., Goulet L., Konz T., Martin F.P. (2018). Menstrual Cycle Rhythmicity: Metabolic Patterns in Healthy Women. Sci. Rep..

[54] Bäckström T., Bixo M., Johansson M., Nyberg S., Ossewaarde L., Ragagnin G., Savic I., Strömberg J., Timby E., van Broekhoven F. (2014). Allopregnanolone and Mood Disorders. Prog. Neurobiol..

[55] Schumacher M., Mattern C., Ghoumari A., Oudinet J.P., Liere P., Labombarda F., Sitruk-Ware R., de Nicola A.F., Guennoun R. (2014). Revisiting the Roles of Progesterone and Allopregnanolone in the Nervous System: Resurgence of the Progesterone Receptors. Prog. Neurobiol..

[56] Sotler R. (2019). Prooxidant Activities of Antioxidants and Their Impact on Health. Acta Clin. Croat..

[57] Reed S.C., Levin F.R., Evans S.M. (2008). Changes in Mood, Cognitive Performance and Appetite in the Late Luteal and Follicular Phases of the Menstrual Cycle in Women with and without PMDD (Premenstrual Dysphoric Disorder). Horm. Behav..

[58] Moher D., Liberati A., Tetzlaff J., Altman D.G. (2009). The PRISMA Group Preferred Reporting Items for Systematic Reviews and Meta-Analyses: The PRISMA Statement. PLoS Med..

