# Supplementary material for: Is Premenstrual Syndrome Associated with Inflammation, Oxidative Stress and Antioxidant Status? A Systematic Review of Case–Control and Cross-Sectional Studies
Source: Antioxidants (Basel). 2021 Apr 14;10(4):604. doi: 10.3390/antiox10040604 (PMC8070917; doi:10.3390/antiox10040604)
Supplement: Supplementary file 1 [file antioxidants-10-00604-s001.pdf]

SUPPLEMENTARY MATERIAL

Is premenstrual syndrome associated with inflammation, oxidative stress and antioxidant status? A systematic review of case-control and cross-sectional studies

Dominika Granda*, Maria Karolina Szmidt, Joanna Kaluza

***Corresponding author:** Dominika Granda, Institute of Human Nutrition Sciences, Warsaw University of Life Sciences – SGGW, 159C Nowoursynowska Str., 02-776 Warsaw, Poland, E-mail: dominika_granda@sggw.edu.pl, Tel: +48 22 59 37 119

Affiliations:

Institute of Human Nutrition Sciences, Warsaw University of Life Sciences–SGGW, 02-776 Warsaw, Poland

Appendix 1. Quality assessment of studies included in the systematic review

The studies included in the systematic review have been evaluated in terms of methodological quality according to a Modified Newcastle-Ottawa Quality Assessment Scale for case-control studies [1] or cross-sectional studies [2].

CASE-CONTROL STUDIES

For case-control studies the maximum of total points was 9; 0 to 3 points indicated – low-quality study, 4 to 6 points – medium-quality study, and 7 to 9 points – high-quality study. Each study was assessed in terms of 3 categories: selection, comparability, and exposure. The criteria, which were taken into consideration while assessing the studies, are presented below.

Selection (maximum 4 points)

(1) Is the case definition adequate?

(a) Yes, with independent validation, in our case we accepted Daily Symptom Record as the best method, but we also accepted other well-described scales like Calendar of Premenstrual Experience (COPE) or Premenstrual Symptoms Screening Tool (PSST) with modifications [**1 point**];

(b) yes, e.g., record linkage or based on self-reports – when patients were only asked whether they define themselves as PMS cases [0 points];

(c) no description [0 points]

(2) Representativeness of the cases:

(a) consecutive or obviously representative series of cases (cases with the outcome of interest over a defined period, all cases in a defined catchment area, all cases in a defined hospital or clinic, group of hospitals, health maintenance organization, or an appropriate sample of those cases) [**1 point**];

(b) potential for selection biases or not stated [0 points].

(3) Selection of controls:

(a) community controls – cases and controls had to be from the same population [one point];

(b) hospital controls [0 points];

(c) no description [0 points].

(4) Definition of controls:

(a) no history of disease (end-point) [**1 point**];

(b) no description of source [0 points].

Comparability (maximum 2 points)

(1) Comparability of cases and controls on the basis of design or analysis:

(a) study controls for age [**1 point**];

(b) study controls for other potentially important factors like body mass index. *We decided not to make strict criteria because factors affecting PMS are still not definitively defined and are the subject of research* [**1 point**].

We accepted statements of no differences between groups or statements that the differences were not statistically significant as sufficient for establishing comparability.

(c) no factors included [0 points]

Exposure (maximum 3 points)

(1) Ascertainment of exposure:

(a) secure record [**1 point**];

(b) structured interview where blind to case/control status – when women did not know what criteria had to be met to be classified into one of the groups [**1 point**];

(c) interview not blinded to case/control status [0 points];

(d) written self-report or medical record only [0 points];

(e) no description [0 points].

(2) Same method of ascertainment for cases and controls:

(a) yes [**1 point**];

(b) no [0 points].

(3) Non-Response rate:

(a) same rate for both groups [**1 point**];

(b) non respondents described [0 points];

(c) rate different and no designation [0 points].

CROSS-SECTIONAL STUDIES

For cross-sectional studies the maximum of total points was 10; 0 to 3 points indicated – low-quality study, 4 to 6 points – medium-quality study, and 7 to 10 points – high-quality study. Each study was assessed in terms of 3 categories: selection, comparability, and outcome. The criteria, which were taken into consideration while assessing the studies, are presented below.

Selection (maximum 5 points)

(1) Representativeness of the sample:

(a) Truly representative of the average in the target population - all subjects or random sampling [**1 point**];

(b) Somewhat representative of the average in the target population - non-random sampling. We accepted studies where the sample included women from various faculties, universities, provinces etc. [**1 point**];

(c) Selected group of users [0 points];

(d) No description of the sampling strategy [0 points].

(2) Sample size

(a) Justified and satisfactory. We accepted every mention, e.g. calculation of sample power [**1 point**];

(b) Not justified [0 points].

(3) Non-respondents

(a) Comparability between respondents and non-respondents characteristics is established, and the response rate is satisfactory [**1 point**];

(b) The response rate is unsatisfactory, or the comparability between respondents and non-respondents is unsatisfactory [0 points];

(c) No description of the response rate or the characteristics of the responders and the non-responders [0 points].

(4) Ascertainment of the exposure (risk factor)

(a) Validated measurement tool – PMS diagnosed by Daily Symptom Record Scale [**2 points**];

(b) Non-validated measurement tool, but the tool is available or described [**1 point**];

(c) No description of the measurement tool [0 points].

Comparability (maximum 2 points)

(1) The subjects in different outcome groups are comparable, based on the study design or analysis. Confounding factors are controlled.

(a) The study controls for the most important factor - age [**1 point**];

(b) The study control for any additional factor [**1 point**];

Outcome (maximum 3 points)

(1) Assessment of the outcome:

(a) Independent blind assessment [**2 points**];

(b) Record linkage – well described scales like Daily Symptom Record Scale, Premenstrual Symptom Screening Test etc. [**2 points**];

(c) Self report [**1 point**];

(d) No description [0 points].

(2) Statistical test

(a) The statistical test used to analyze the data is clearly described and appropriate, and the measurement of the association is presented, including confidence intervals and the probability level (p value) [**1 point**];

(b) The statistical test is not appropriate, not described or incomplete [0 points].

References:

1. Wells, G.A.; Shea, B.; O'Connell, D.; Peterson, J.; Welch, V.; Losos, M.; Tugwell, P. The Newcastle-Ottawa Scale (NOS) for Assessing the Quality of Nonrandomised Studies in Meta-Analyses Available online: http://www.ohri.ca/programs/clinical_epidemiology/oxford.asp (accessed on 23/02/2021)
2. Modesti, P.A.; Reboldi, G.; Cappuccio, F.P.; Agyemang, C.; Remuzzi, G.; Rapi, S.; Perruolo, E.; Parati, G.; ESH Working Group on CV Risk in Low Resource Settings Panethnic Differences in Blood Pressure in Europe: A Systematic Review and Meta-Analysis. PLOS ONE 2016, 11, e0147601, doi:10.1371/journal.pone.0147601.
